# Supplementary material for: ES-SCLC Patients with PD-L1+ CTCs and High Percentages of CD8+PD-1+T Cells in Circulation Benefit from Front-Line Immunotherapy Treatment
Source: Biomedicines. 2024 Jan 10;12(1):146. doi: 10.3390/biomedicines12010146 (PMC10813758; doi:10.3390/biomedicines12010146)
Supplement: Supplementary file 1 [file biomedicines-12-00146-s001.zip › biomedicines-2785595-supplementary.pdf]

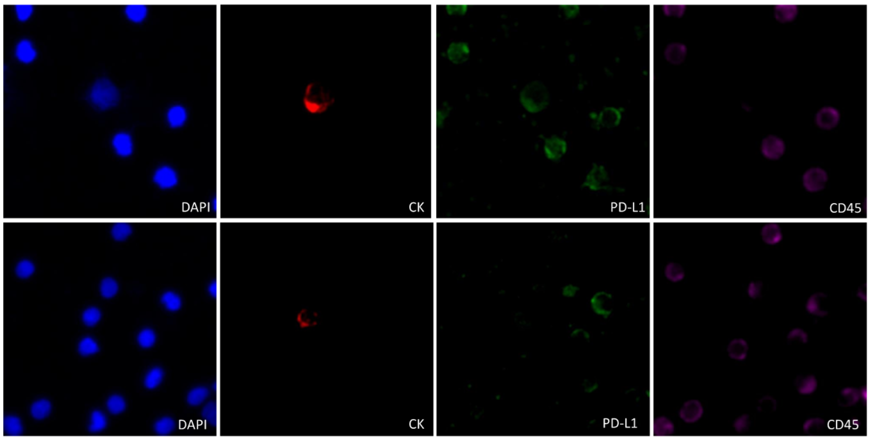

Figure S1: Representative pictures of stained CTCs.

Table S1. Number of CTCs detected for each patient.

		Number of CTCs			% of CTCs	
**A/A**	**Stage**	**CK+**	**CK+PDL1+CD45-**	**CK+PDL1-CD45-**	**%CK+PDL1+CD45-**	**%CK+PDL1-CD45-**
**1**	LIMITED	5	3	2	60,00	40,00
**2**	EXTENSIVE	0	0	0	0,00	0,00
**3**	EXTENSIVE	0	0	0	0,00	0,00
**4**	EXTENSIVE	3	0	3	0,00	100,00
**5**	EXTENSIVE	0	0	0	0,00	0,00%
**6**	EXTENSIVE	0	0	0	0,00	0,00
**7**	EXTENSIVE	0	0	0	0,00	0,00
**8**	EXTENSIVE	0	0	0	0,00	0,00
**9**	EXTENSIVE	0	0	0	0,00	0,00
**10**	EXTENSIVE	0	0	0	0,00	0,00
**11**	EXTENSIVE	0	0	0	0,00	0,00
**12**	EXTENSIVE	1	1	0	0,00	0,00
**13**	LIMITED	0	0	0	0,00	0,00
**14**	EXTENSIVE	0	0	0	0,00	0,00
**15**	EXTENSIVE	0	0	0	0,00	0,00
**16**	EXTENSIVE	19	9	10	47,37	52,63
**17**	EXTENSIVE	2	1	1	50,00	50,00
**18**	EXTENSIVE	0	0	0	0,00	0,00
**19**	EXTENSIVE	0	0	0	0,00	0,00
**20**	EXTENSIVE	1	1	0	100,00	0,00
**21**	EXTENSIVE	0	0	0	0,00	0,00
**22**	EXTENSIVE	1	1	0	100,00	0,00
**23**	EXTENSIVE	0	0	0	0,00	0,00
**24**	EXTENSIVE	0	0	0	0,00	0,00
**25**	LIMITED	0	0	0	0,00	0,00
**26**	EXTENSIVE	1	1	0	100,00	0,00
**27**	EXTENSIVE	1	1	0	100,00	0,00
**28**	EXTENSIVE	2	2	0	100,00	0,00
**29**	EXTENSIVE	0	0	0	0,00	0,00
**30**	EXTENSIVE	1	1	0	100,00	0,00
**31**	EXTENSIVE	1	0	1	0,00	100,00
**32**	LIMITED	0	0	0	0,00	0,00
**33**	EXTENSIVE	0	0	0	0,00	0,00
**34**	EXTENSIVE	2	2	0	100,00	0,00
**35**	EXTENSIVE	6	5	1	83,33	16,67
**36**	EXTENSIVE	0	0	0	0,00	0,00
**37**	LIMITED	1	0	1	0,00	100,00
**38**	EXTENSIVE	0	0	0	0,00	0,00
**39**	EXTENSIVE	70	13	57	18,57	81,43
**40**	EXTENSIVE	0	0	0	0,00	0,00
**41**	LIMITED	0	0	0	0,00	0,00
**42**	EXTENSIVE	0	0	0	0,00	0,00
**43**	EXTENSIVE	23	15	8	65,22	34,78

Table S2. Associations of CTCs and clinical outcome of treatment naïve ES-SCLC patients.

			**Progresion Free Survival**			**Overall Survival**		
**CTCs**	**Groups**	**N of patients**	**Median (days)**	**95% HR CI**	**p value**	**Median (days)**	**95% CI**	**p value**
**CK+CD45-**	>1	8	176	0.303 to 1.612	0.401	260	0.277 to 2.042	0.311
	<1	29	163			232		
**CK+CD45-**	>3	5	167	0.586 to 6.586	0.273	180	0.534 to 5.820	0.352
	<3	32	167			261		
**CK+CD45-**	PD-L1+	13	172	0.355 to 1.772	0.572	271	0.213 to 1.4	0.207
	other	24	158			217		

CTC: circulating tumor cell; CK : Cytokeratin; PD-L1: programmed death ligand 1; HR: hazard ratio, CI: confidence interval estimate.

Table S3. Prognostic significance of PD-L1$^+$ CTCs or CK+ CTCs and CD3+CD8$^+$ PD-1$^+$ T-cells in patients with ES-SCLC.

	**Corelations**	**95% HR CI**	**p value**	**95% CI**	**p value**
**CK+CTCs**	**Group A vs Group B**	0.2972 to 3.230	0.973	0.2195 to 5.599	0.900
	**Group A vs Group C**	0.01322 to 1.683	0.123	0.009 to 1.424	0.092
	**Group A vs Group D**	0.1641 to 1.299	0.143	0.8630 to 1.635	0.061
	**Group B vs Group C**	0.1580 to 3.667	0.733	0.07137 to 5.088	0.641
	**Group B vs Group D**	0.2163 to 2.231	0.540	0.1321 to 2.354	0.426
	**Group C vs Group D**	0.2758 to 4.800	0.847	0.1500 to 2.266	0.436
**CK+CTCs**	**Group A vs Group B**	0.3429 to 3.093	0.958	0.1755 to 3.838	0.801
	**Group A vs Group D**	0.1726 to 1.177	0.103	0.099 to 0.8197	**0.019**
	**Group B vs Group D**	0.208 to 1.614	0.297	0.1766 to 2.111	0.435

CTC: circulating tumor cell; CK : Cytokeratin; PD-1: programmed cell death receptor 1; PD-L1: programmed death ligand 1; HR: hazard ratio, CI: confidence interval estimate ; group A high CD3+CD8+PD-1+ T-cells (roc cut off) with PD-L1+CTCs (n=13) or CK+ CTCs (n= 15) , group B: high CD3+CD8+PD-1+ T-cells (roc cut off) without PD-L1+CTCs (n=12) or CK+ CTCs (n= 10) , group C: low CD3+CD8+PD-1+ T-cells (roc cut off) with PD-L1+CTCs (n=1) or or CK+ CTCs (n= 2) and group D: low CD3+CD8+PD-1+ T-cells (roc cut off) without PD-L1+CTCs (n=11) or CK+ CTCs (n= 10). Analysis of group C: low CD3+CD8+PD-1+ T-cells (roc cut off) with PD-L1+CTCs (n=1) was not performed as there was only one patient.

Table S4. Cox regression analysis.

Risk Factor	Parameter Estimate	P-Value	Hazard Ratio (HR) (95% CI for HR)
Age, years	0,03028	0,5925	0,9288 to 1,167
Liver Metastasis	1,457	0,1939	0,6514 to 84,70
Brain Metastasis	-0,7043	0,4463	0,06470 to 2,640
Lung Metastasis	-1,082	0,189	0,06282 to 1,754
Bone Metastasis	-1,678	0,3272	0,004018 to 6,483
Tumor diameter	-0,4144	0,7337	0,07486 to 14,32
Smoking (py)	0,4356	0,578	0,3309 to 7,781
